# Internal Ribosome Entry Segment Activity of *ATXN8* Opposite Strand RNA

**DOI:** 10.1371/journal.pone.0073885

**Published:** 2013-09-11

**Authors:** I-Cheng Chen, Hsuan-Yuan Lin, Ya-Chin Hsiao, Chiung-Mei Chen, Yih-Ru Wu, Hsin-Chieh Shiau, Yu-Fang Shen, Kuo-Shiu Huang, Ming-Tsan Su, Hsiu-Mei Hsieh-Li, Guey-Jen Lee-Chen

**Affiliations:** 1 Department of Life Science, National Taiwan Normal University, Taipei, Taiwan; 2 Department of Neurology, Chang Gung Memorial Hospital, Chang-Gung University College of Medicine, Taipei, Taiwan; National Center of Neurology and Psychiatry, Japan

## Abstract

Spinocerebellar ataxia type 8 (SCA8) involves the expansion of CTG/CAG repeats from the overlapping ataxin 8 opposite strand (*ATXN8OS*) and ataxin 8 (*ATXN8*) genes located on chromosome 13q21. Although being transcribed, spliced and polyadenylated in the CTG orientation, *ATXN8OS* does not itself appear to be protein coding, as only small open reading frames (ORFs) were noted. In the present study we investigated the translation of a novel 102 amino acids containing-ORF in the *ATXN8OS* RNA. Expression of chimeric construct with an in-frame ORF-EGFP gene demonstrated that *ATXN8OS* RNA is translatable. Using antiserum raised against ORF, *ATXN8OS* ORF expression was detected in various human cells including lymphoblastoid, embryonic kidney 293, neuroblastoma IMR-32, SK-N-SH, SH-SY5Y cells and human muscle tissue. The biological role of the *ATXN8OS* ORF and its connection to SCA8 remains to be determined.

## Introduction

The spinocerebellar ataxias (SCAs) comprise a heterogeneous group of disorders involving progressive degeneration of the cerebellum, brainstem, and spinal tract [1]. Of all SCAs, SCA type 8 (SCA8) presents a molecular trait that distinguishes it from other dominant ataxias: its involving a CTG repeat expansion in the *ATXN8OS* (ataxin 8 opposite strand) gene and a CAG repeat expansion in the overlapping *ATXN8* (ataxin 8) gene [2]. In the CTG direction, *ATXN8OS* expresses spliced and polyadenylated untranslated transcripts in various brain tissues [3]. In the CAG direction, the expanded *ATXN8* encodes a polyglutamine expansion protein [4] known to be pathogenic in other polyglutamine disorders.

The pathogenesis of SCA8 appears to be complex. In addition to polyglutamine expansion protein in the CAG direction, other plausible mechanisms related to the transcripts in the CTG direction were also proposed. Firstly, in a *Drosophila* model, the ectopically expressed *ATXN8OS* RNA interacted with RNA binding proteins to lead to late-onset, progressive degeneration in the photoreceptor and pigment cells of flies [5], supporting a RNA gain-of function mechanism [6]. Secondly, partial loss of *Klhl1* function with targeted deletion of a single *Sca8* ataxia locus allele (including to overlapped *KLHL1* gene) in mice leads to degeneration of Purkinje cell function [7], indicating an anti-sense RNA interference mechanism. Our recent study using a cellular model of *ATXN8OS* also revealed that SCA8 larger triplet expansion alters histone modification and induces RNA foci [8]. RNA foci were also seen in SCA8 patient and mouse brains with MBNL1 protein colocalized with these RNA foci [9].

**Figure 1:**
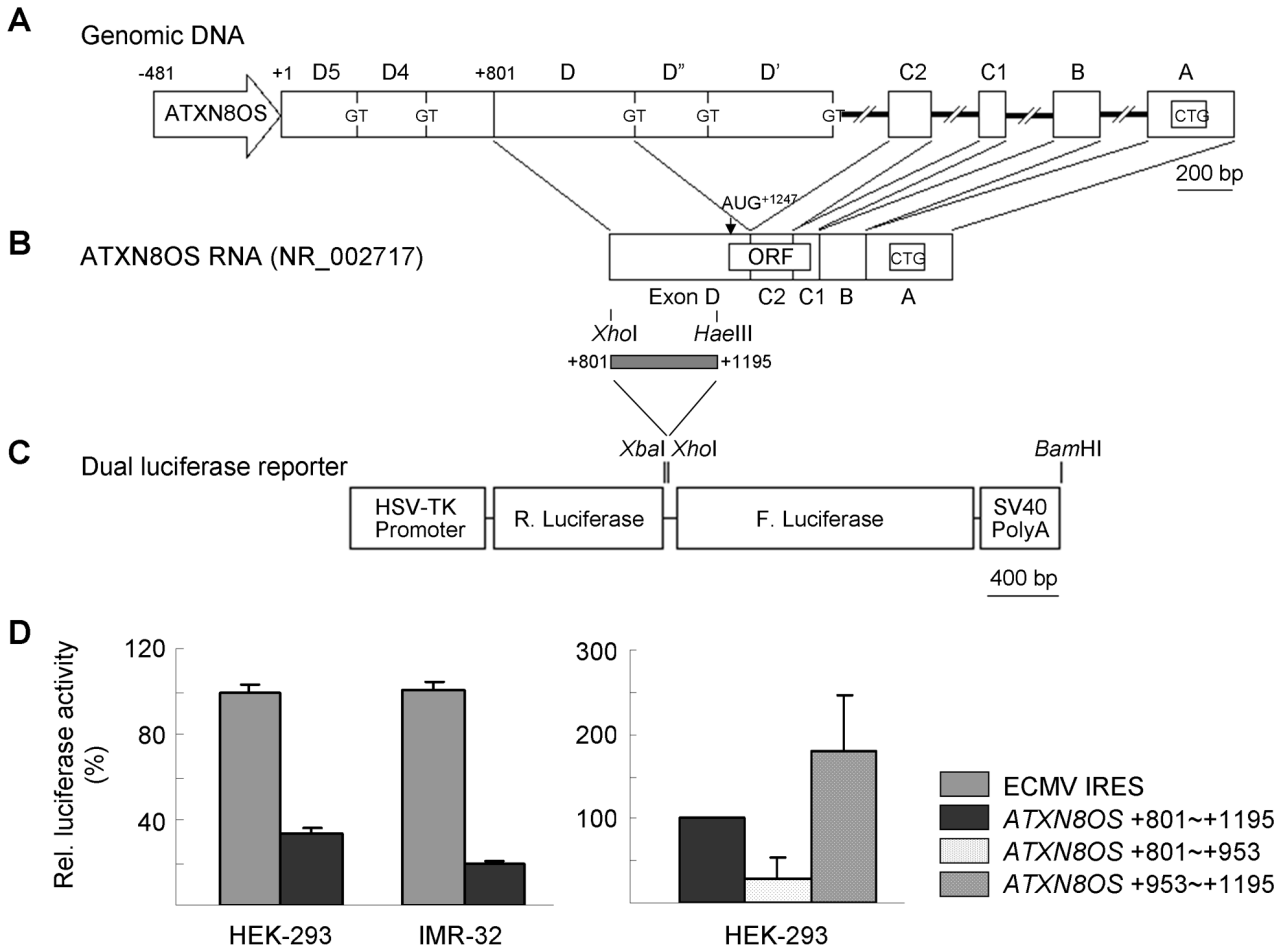
IRES activity of the *ATXN8OS* transcript. (A) *ATXN8OS* organization with promoter (open arrow), exons (open boxes) and functional splice donor sequences (GT) of D exons (D5, D4, D, D″ and D′) indicated. The CTG repeat tract is located in exon A. Transcription start site of exon D5 and exon D are represented by +1 and +801, respectively. (B) *ATXN8OS* RNA (NR_002717) generated from the splicing events represented by the wavy lines. The putative ORF initiated from AUG^+1247^ is indicated by the open boxes inside the RNA. The restriction enzymes and the cutting sites used to generate +801∼+1195 cDNA fragment of *ATXN8OS* are shown on the bottom of the cDNA. (C) The dual luciferase reporter plasmid had *Renilla* luciferase and firefly luciferase genes between the TK promoter and polyadenylation signal. The locations of *Xba*I, *Xho*I and *Bam*HI sites used for construction are shown on the top. (D) Relative luciferase activities generated by dual luciferase constructs with ECMV IRES and *ATXN8OS* +801∼+1195 cDNA fragment in HEK-293 and IMR-32 cells. Forty-eight hours following transfection, cells were harvested and luciferases activities were measured. IRES activity is expressed as percentages of the activity of the ECMV IRES, which was set at 100%. In addition, relative luciferase activities with *ATXN8OS* +801∼+953 and +953∼+1195 cDNA fragments were measured in HEK-293 cells, with IRES activity of +801∼+1195 set at 100%. Each value is the mean ± SD of three independent experiments each performed in duplicate.

Although being apparently non-coding [3], a 102 amino acid-containing open reading frame (ORF) exists. The ORF is 446 nucleotides (according to NR_002717) or 1246 nucleotides (according to [10]) from the 5′ end of *ATXN8OS* RNA (Fig. 1A). In eukaryotes, translation initiation involves recruitment of ribosomal subunits at either the 5′ m7G cap structure or at an internal ribosome entry site (IRES). In cap-dependent mechanism, the initiation codon is located some distance downstream for most mRNAs, requiring ribosomal movement to this site, either linear or going around segments of the 5′ leader to reach the initiation codon [11]. The cap-independent mechanism requires the formation of a complex RNA structural element termed IRES and the presence of *trans*-acting factors [12]. As a result, the ribosome entry window attains an unstructured conformation and in doing so facilitates ribosome recruitment. In addition, non-AUG triplets may be used as translation initiators for gene expression [13], [14]. In this study we firstly examined the cap independent IRES activity in the *ATXN8OS* RNA using a dual luciferase reporter assay. Then we fused the *ATXN8OS* ORF in-frame with an EGFP tag to investigate if the *ATXN8OS* ORF could be translated using cell culture studies. The ORF expression was validated in human lymphoblastoid, neuroblastoma, embryonic kidney cells and muscle tissue using ORF antiserum. The translation of *ATXN8OS* ORF was further examined by tandem MS determination.

## Results

### IRES Activity of *ATXN8OS* RNA

Despite being apparently non-coding [3], a 102 amino-acid ORF (AUG^+1247^) was noted in the *ATXN8OS* transcripts (Fig. 1B). To investigate if this ORF can be translated via a cap independent IRES activity, we constructed a dicistronic vector pRF in which firefly luciferase was placed after the *Renilla* luciferase (Fig. 1C). The expression construct was under the control of the HSV-TK promoter. Sequence upstream of *ATXN8OS* ORF (+801∼+1195; [10]) was inserted into the intercistronic region of the pRF. The IRES from the encephalomyocarditis virus (ECMV) [15] was inserted as a positive control. When the expressed luciferase level of the ECMV IRES was set as 100%, the +801∼+1195 fragment directed firefly luciferase synthesis to a level of 33.7% and 19.6%, respectively, in HEK-293 and IMR-32 cells as compared to the ECMV IRES sequence (Fig. 1D, left). When the +801∼+1195 fragment was further subdivided into +801∼+953 and +953∼+1195, levels of 29.9% and 180.8%, respectively, in HEK-293 cells was observed as compared to the +801∼+1195 fragment sequence (Fig. 1D, right). The result suggests the possible IRES activity existing in the region upstream of *ATXN8OS* ORF.

### *ATXN8OS* ORF Expression

**Figure 2:**
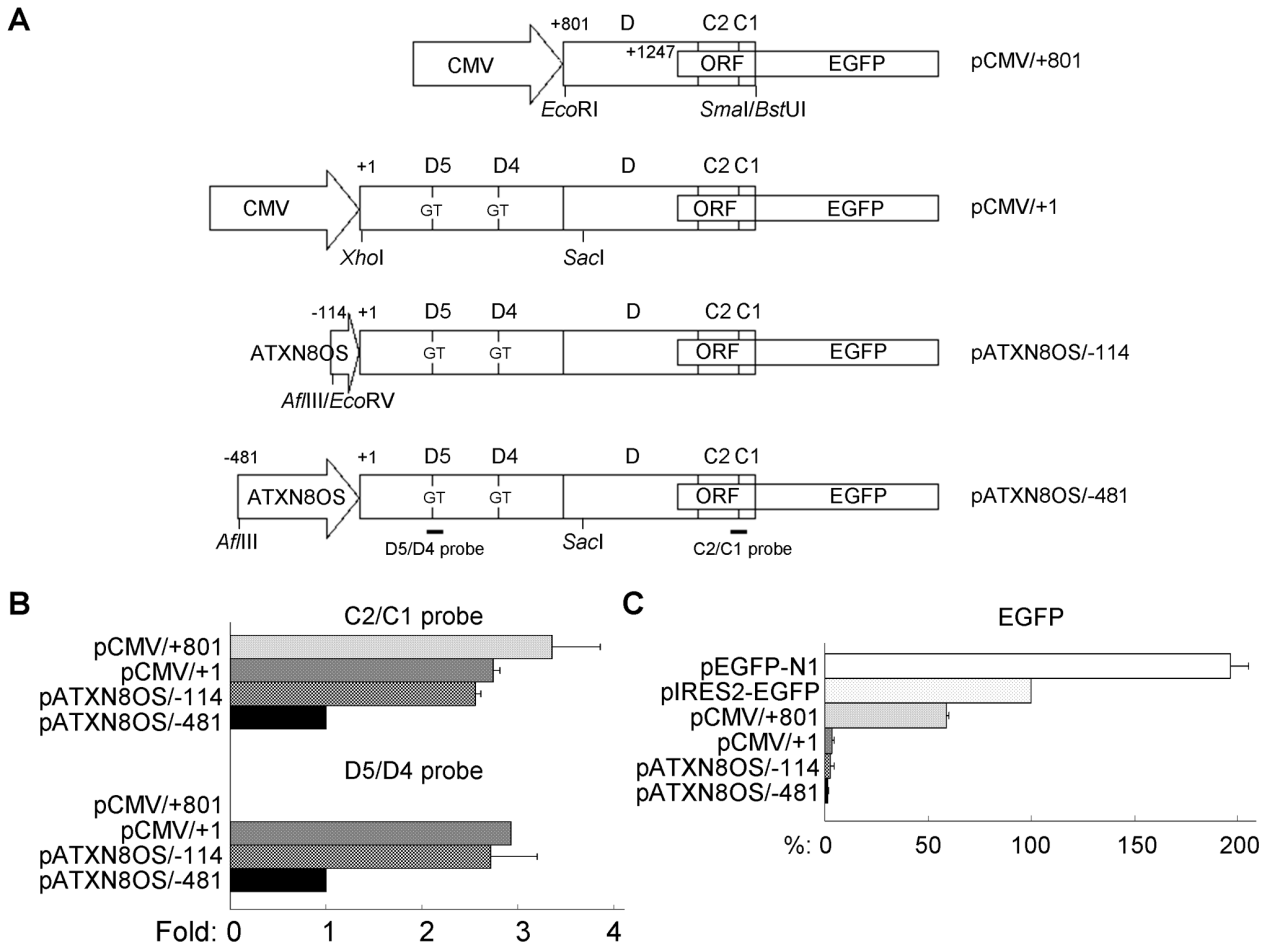
Transient expression of *ATXN8OS* ORF-EGFP constructs in HEK-293 cells. (A) ORF-EGFP constructs. A 752-bp cDNA fragment containing exon D, C2 and portion of C1 was inserted into pEGFP-N1 MCS so that *ATXN8OS* ORF was fused in-frame with the EGFP gene to generate pCMV/+801. A +1∼+800 *ATXN8OS* fragment was inserted between CMV promoter and exon D of pCMV/+801 to generate pCMV/+1. In pATXN8OS/−114 and/−481, 114 and 481-bp *ATXN8OS* promoter fragments was used to replace the CMV promoter in pCMV/+1. (B) Real-time PCR quantification of ORF-EGFP RNA level relative to endogenous *HPRT1* RNA. To normalize, expression level in pATXN8OS/−481 transfected cells is set as 1.0. (C) FACS analysis of EGFP fluorescence. Levels of EGFP were expressed as percentages of pIRES2-EGFP, which was set at 100%. Each value is the mean ± SD of three independent experiments each performed in duplicate.

To investigate if indeed the *ATXN8OS* ORF could be translated, we cloned the *ATXN8OS* cDNA (NR_002717) and in-frame fused an EGFP tag to the C terminal of the *ATXN8OS* ORF (Fig. 2A, pCMV/+801). The transcripts made from this construct will be initiated from exon D (+801). As the promoter region upstream of exon D5 was identified by comparing human and mouse genomic DNA sequences flanking the 5′ end of the transcripts [10], *ATXN8OS* gene sequence +1∼+800 were included in construct pCMV/+1 so that transcripts made will be initiated from exon D5 (+1). In constructs pATXN8OS/−481 and pATXN8OS/−114, proximal *ATXN8OS* promoter fragments −481∼−1 and −114∼−1 were used to drive *ATXN8OS* expression to mimic the *in vivo* situation.

The constructs were transiently expressed into HEK-293 cells. After two days ORF-EGFP RNA levels were measured by real-time PCR quantification using *ATXN8OS*-specific probe C2/C1 and primers. As shown in Fig. 2B, when the expressed level in pATXN8OS/−481 cells was set as 1.0, ORF-EGFP RNA levels for transcripts initiated from +801 (pCMV/+801) versus transcripts initiated from +1 (pCMV/+1, pATXN8OS/−481 and pATXN8OS/−114) were 3.4 and 1.0∼2.7, respectively. Similar 1.0∼2.9 range of ORF-EGFP RNA levels for transcripts initiated from +1 (pCMV/+1, pATXN8OS/−481 and pATXN8OS/−114) were also observed using *ATXN8OS* D5/D4 probe (Fig. 2B).

The EGFP fluorescence was evaluated by FACS analysis. As shown in Fig. 2C, compared to the pIRES2-EGFP (cap-independent EGFP expression, 100%), 196.5% EGFP fluorescence was seen in cells transfected with pEGFP-N1 (cap-dependent EGFP expression). For the ORF-EGFP constructs, 1.4∼59.0% EGFP fluorescence was seen as compared to the IRES-dependent EGFP fluorescence (pIRES2-EGFP). Transcripts initiated from +801 (pCMV/+801; 59.0% of pIRES2-EGFP) expressed 17∼42 fold EGFP fluorescence compared to transcripts initiated from +1 (pCMV/+1, pATXN8OS/−481 and pATXN8OS/−114; 1.4∼3.5% of pIRES2-EGFP).

**Figure 3:**
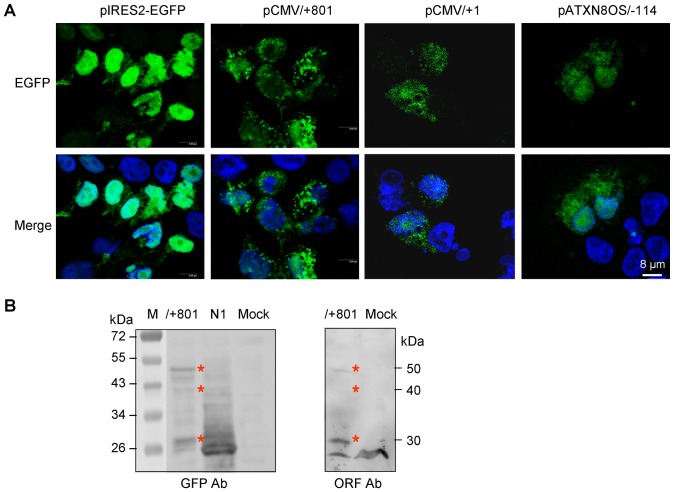
Protein expression of *ATXN8OS* ORF-EGFP fusion protein in HEK-293 cells. (A) Confocal images of cells expressing EGFP and ORF-EGFP. Cells were transfected with pIRES2-EGFP, pCMV/+801, pCMV/+1 and pATXN8OS/−114 for two days. After counterstained nuclei with DAPI, cells were examined using a confocal microscope for GFP (green) and DAPI (blue) dual fluorescent imaging. (B) Western blot analysis of cells expressing ORF-EGFP and EGFP proteins. Cells were transfected with pCMV/+801, pEGFP-N1, or mock-transfected. After two days, cell lysates were prepared and proteins analyzed with anti-GFP antibody or anti-ORF antiserum.

To visualize the expression of ORF-EGFP protein, confocal microscopic examination of GFP fluorescence was carried out after transfection of pIRES2-EGFP, pCMV/+801, pCMV/+1 and pATXN8OS/−114 constructs into HEK-293 cells. As shown in Fig. 3A, strong GFP fluorescence was distributed diffusely in pIRES2-EGFP-transfected cells. With pCMV/+801 construct, small and dispersed granules appeared mainly in the cytoplasm, in addition to showing diffuse cytoplasm expression. Cells transfected with pCMV/+1 or pATXN8OS/−114 gave sparse granules and weak, diffuse GFP fluorescence.

To examine the expressed ORF-EGFP protein, GST-ORF (*S. japonicum* GST from pGEX plasmid) fusion protein was prepared as antigen to raise antiserum in rabbit. Western blot immunostaining with GFP antibody or ORF antiserum was performed. As shown in Fig. 3B, similar proteins (40 and 30 kDa) were detected in cells transfected with pCMV/+801. Whereas the weakly expressed 40 kDa protein may represent the predicted ORF-EGFP protein (AUG^+1247^ start, 348 amino acids with MW of 39472; ExPASy: http://web.expasy.org/compute_pi/), the 30 kDa protein apparently differs from the predicted. The 30 kDa protein may be initiated from a downstream in-frame AUG codon (AUG^+1490^ start, 267 amino-acid fusion protein, MW 30061). A lager protein around 50 kDa was also noted by the Western blot either probing with GFP antibody or ORF antiserum. The existence of this 50 kDa protein indicated that ORF-EGFP protein may be translated from the sequence upstream of AUG^+1247^.

### ORF Immunodetection

**Figure 4:**
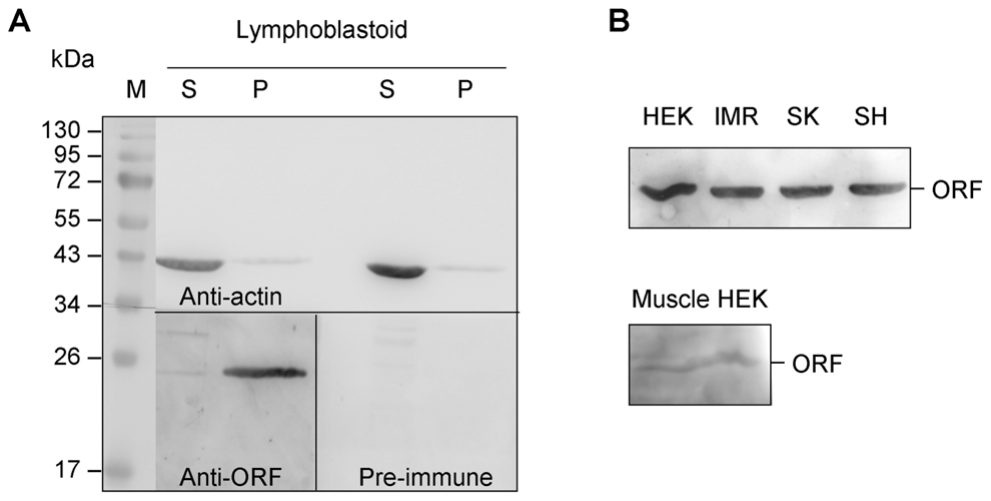
ORF protein expression in human cells. (A) Western blot analysis of lymphoblastoid cell proteins. With urea lysis buffer, cells lysates were divided into soluble fraction (S) and pellet (P). The insoluble pellets were then dissolved in SDS buffer. Proteins from soluble and pellet fractions were analyzed with actin antibody, ORF antiserum or pre-immune serum. (B) Western blot analysis of human embryonic kidney cells (HEK-293), neuroblastoma (IMR-32, SK-N-SH and SH-SY5Y) cells and muscle tissue. Proteins from urea lysis buffer-insoluble pellets were analyzed with ORF antiserum.

To validate if indeed *ATXN8OS* ORF is expressed in human cells, ORF antiserum was used to detect the possible endogenous ORF protein. As we hardly detected ORF protein in RIPA-soluble fraction and also the predicted 102 amino acids *ATXN8OS* ORF protein has a 62.9% chance of insolubility when overexpressed in *E. coli* (http://www.biotech.ou.edu/), urea lysis buffer was used for lymphoblastoid protein extraction since the average molecular weight of proteins that dissolve exclusively in urea buffer is up to 60% higher than in RIPA buffer [16]. On Western blot staining with ORF antiserum, while no specific polypeptide was detected with pre-immune serum, an unexpected 23 kDa protein was detected in insoluble pellet fraction (Fig. 4A). The same 23 kDa protein was also observed in urea buffer-insoluble pellet fraction prepared from embryonic kidney 293 cells, neuroblastoma IMR-32, SK-N-SH, SH-SY5Y cells and human muscle tissue (Fig. 4B).

### ORF Identification

**Figure 5:**
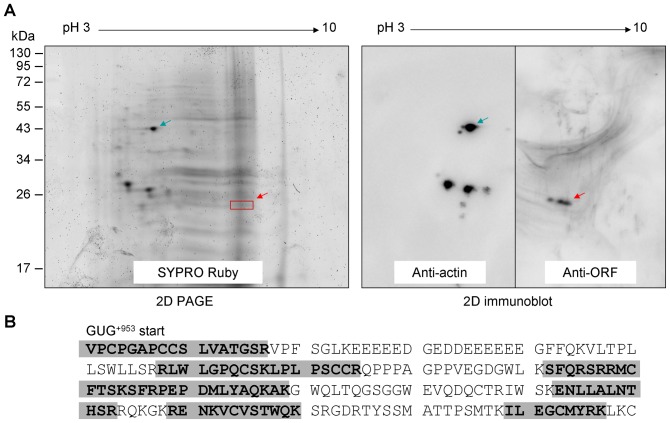
ORF protein identification. (A) 2D PAGE image and 2D immunoblot. Proteins from urea lysis buffer-insoluble pellets were separated using 2D gels for SYPRO Ruby staining (left, 2D PAGE) and ORF antiserum or actin antibody staining (right, 2D immunoblot). The 2D PAGE map was compared to the 2D immunoblot to obtain ORF-specific spots (red arrow). (B) *ATXN8OS* ORF amino acid sequences initiated from GUG^+953^. The ORF-specific spots were analyzed and MS/MS data were searched in a database containing theoretical trypsinized fragments of 23-kDa ORF protein initiated at GUG^+953^ codon. Six matched peptides determined by LC-MS/MS were marked in boldface.

To identify the endogenous *ATXN8OS* ORF protein, lymphoblastoid proteins from urea buffer-insoluble pellet fraction were subjected to 2D PAGE and 2D immunoblot (Fig. 5A). The identity of the three ORF-specific spots was determined using LC-MS/MS and Mascot data search in a database set up for the predicted ORF. As shown in Fig. 5B, six matched peptide with sequence coverage of 47% was obtained, including the N-terminal peptide VPCPGAPCCS LVATGSR which can only be generated from translation start from GUG^+953^ due to the stop codon UGA existing upstream of GUG^+953^.

## Discussion

The *ATXN8OS* gene was isolated from a single sample directly, using the RAPID cloning method [3], [17]. Sequence analysis revealed that the expansion consisted of a stretch of 11 CTA repeats followed by 80 CTG repeats. Analysis of this sequence did not reveal any possible spliced isoform possessing an ORF to extend through the expansion in either direction. Therefore, SCA8 was first proposed to be caused by an RNA gain-of-function mechanism [6]. In this study, we used dual luciferase assay to demonstrate that *ATXN8OS* RNA +801∼+1195 had IRES activity (Fig. 1). As *ATXN8OS* ORF detected in human cells was predicted to be translated from GUG^+953^ (Fig. 5), the IRES activity of +801∼+953 was compared with that of +953∼+1195. To our surprise, the +953∼+1195 fragment showed higher IRES activity while less IRES activity was observed from +801∼+953 fragment (Fig. 1). The presence of a 12 amino-acid ORF (AUG^+^890∼UAG^+926^) within +801∼+953 fragment may explain the reduced amount of translation that occurs from the downstream firefly luciferase cistron. Similar translation read-through of cellular transcripts can be seen with human angiotensin II type 1 receptor (AGTR1) mRNA (IRES name: AT1R_var3; http://www.iresite.org/IRESite_web.php?page=browse_cellular_transcripts) [18], [19]. Accordingly, the enhancing IRES activity observed with +953∼+1195 fragment may be explained by the removal of inhibition derived from the small ORF’s translation. As cap-independent mechanism requires the formation of a complex RNA structural element and the presence of *trans*-acting factors, it is also likely that some inhibitory factors may exist within +801∼+953 fragment and regulate *ATXN8OS* RNA IRES activity. The *trans*-acting factors are worthy to be further identified to investigate the translation mechanism of *ATXN8OS* RNA.

In our study, the predicted translation start GUG^+953^ was within the *ATXN8OS* IRES region +801∼+1195, which is different from the general concept that putative IRES sequences are located in a close proximity to the 5′ coding region of the genes. Nevertheless, the putative IRES region (+33∼+362, according to NM_004835) of *hAT1R-C* v3 mRNA also overlapped with translation of AGTR1 isoform II N-terminal 35 amino acids (AUG^+258^∼AAA^+360^) (http://www.iresite.org/IRESite_web.php?page=view&entry_id=84), which could support our finding.

Whereas transcripts initiated from +801 and +1 displayed similar range of ORF RNA level, very different range of EGFP fluorescence was seen between transcripts initiated from +801 and +1 (Fig. 2). Unknown proteins or factors binding to *ATXN8OS* RNA +1∼+800 to down-regulate *ATXN8OS* ORF translation are also worthy to be further investigated.

When the expression of ORF-EGFP protein was visualized by confocal microscopic examination, more or less small and dispersed cytosolic granules were observed (Fig. 3A), correlated with ORF RNA (Fig. 2B) and EGFP fluorescence (Fig. 2C) levels. The cytosolic expression of GFP-tagged ORF was also supported by Western blotting of stepwise isolation of cytoplasmic and nuclear fractions and confocal microscopy examination of images from continuous focal planes (data not shown).

Using antiserum raised against ORF, the expression of *ATXN8OS* ORF was validated in various human cells and muscle tissue (Fig. 4). The observed 23 kDa ORF protein is likely initiated from the GUG^+953^ (Fig. 5). A 50 kDa protein was also detected with EGFP antibody or ORF antiserum when ORF-EGFP fusion protein was transiently expressed in 293T cells (Fig. 3). As cellular and viral mRNAs can initiate from non-AUG codons that differ from AUG by just one nucleotide [14], the 23 kDa ORF protein or 50 kDa fusion protein was predicted to be initiated from the same upstream in-frame GUG codon (GUG^+953^ start, 200 amino-acid ORF, predicted MW 22669 or 446 amino-acid ORF-EGFP, predicted MW 50324).

Utilization of alternative non-AUG translation initiation codons has been demonstrated with increasing frequency in mammalian species, in addition to initiating at a downstream in-frame AUG codon [20]. Translation initiation on such mRNAs results in the synthesis of proteins harboring different amino terminal domains potentially conferring on these isoforms distinct functions. As alternative initiation sites are utilized for the synthesis of proteins that regulate biological processes in health and disease [21]–[23], the biological meaning of the *ATXN8OS* ORF protein and its role in the pathogenesis of SCA8 remains to be determined.

Previously bioinformatics analyses demonstrated that distinct consensus sequences (at −7 and −6 positions), upstream AUGs, 5′-UTR sequence length, G/C ratio and IRES secondary structure are important for categorizing mRNAs as those with and without alternative translation initiation sites [24]. Among these properties, 5′-UTR of the alternative translation initiation sites showed conservation of G/C at the −6 position and C at the −7 position. In contrast, the AUG initiation sites showed consensus at position −3 for A/G and position +4 for G/A [24], [25]. The *ATXN8OS* ORF GUG initiation codon has conserved C at the −7 position but less abundance U at the −6 position, the downstream in-frame AUG codon has conserved A at the −3 position but also less abundance U at the +4 position. Although not well conserved at the −6 position, the conserved C at the −7 position and other un-analyzed properties may support the use of the second most common alternative translation initiation GUG site [23] for the translation of *ATXN8OS* ORF protein.

In summary, our study indicated that the *ATXN8OS* putative ORF protein could be translatable and may be expressed via a naturally occurring non-AUG start codon. The biological role of *ATXN8OS* ORF and its connection to SCA8 are deserving of further investigation.

## Materials and Methods

### Ethics Statement

This study was performed according to a protocol approved by the institutional review boards of Chang Gung Memorial Hospital, and all examinations were performed after obtaining written informed consents.

### Dual Luciferase Reporter Constructs

The 1.3-kb *ATXN8OS* cDNA containing exons D, C2, C1, B, and A [26] (Fig. 1B) was cloned as described [8]. The *ATXN8OS* cDNA were then cloned into the *Eco*RI site of pEGFP-N1 (Clontech). To construct a dual luciferase reporter, a 76-bp *Xba*I-*Bam*HI polylinker region of pcDNA3 was first added between the *Xba*I and *Bam*HI sites of phRL-TK vector (Promega) to introduce a *Xho*I site as well as remove the SV40 late poly(A) region. Then a 1972-bp *Xho*I-*Bam*HI fragment containing the firefly luciferase gene and the SV40 late poly(A) signal from pGL3-Basic vector (Promega) was placed between the *Xho*I and *Bam*HI sites of the modified phRL-TK vector. The resulting dual luciferase reporter plasmid had *Renilla* luciferase and firefly luciferase genes between the TK promoter and polyadenylation signal (Fig. 1C). The *ATXN8OS* cDNA in pEGFP-N1 was restricted with *Xho*I and *Hae*III and the blunted cDNA fragment (+801∼+1195) was placed in the blunted *Xho*I site between the two luciferase genes. The sense and antisense primers used for *ATXN8OS* +801∼+953 and +953∼+1195 cDNA amplification were 5′-GCGCCGAATTCATCCTTCACCTGTT and 5′-CAAAAGCTTCTCAGCAGCCAGCCA, and 5′-GGTTAGAATTCGTGCCCTGCCCAGG and 5′-AAATAAGCTTCCCGGCGGGGGGA, respectively (*Eco*RI and *Hin*dIII sites underlined). The resulting PCR products were cloned, sequenced and restricted with *Eco*RI and *Hin*dIII to replace the +801∼+1195 fragment in dual luciferase reporter plasmid. The 632-bp blunted *Xho*I-*Msc*I IRES fragment from pIRES2-EGFP (Clontech) was inserted between the two luciferase genes as a positive control.

### Luciferase Reporter Assay

Human embryonic kidney HEK-293 and neuroblastoma IMR-32 cells were cultivated in Dulbecco’s modified Eagle’s medium (DMEM) containing 10% FBS. Cells were plated into 12-well dishes (2×10^5^/well), grown for 20 hr and transfected by the lipofection method (GibcoBRL) with the test dual luciferase reporter plasmid (1.5 µg/well). The cells were grown for 48 hr. Then cell lysates were prepared and luciferase activity was measured by a luminometer using a dual luciferase assay system (Promega). The IRES activity was directly measured by the ratio of the firefly luciferase level to the *Renilla* luciferase level. For each construct, three independent transfection experiments were performed.

### *ATXN8OS* ORF-EGFP Constructs

The ORF translation termination sequence in C1 exon was removed and a *Sma*I restricted site (underlined) was added by PCR using primer 5′-GCGCCCGGGACACTTCAACTTCCTATACATACA and cloned into pGEM-T Easy (Promega). The *Eco*RI (in MCS of pGEM-T Easy vector)-*Sma*I fragment containing *ATXN8OS* ORF was in-frame fused to the EGFP gene in the pEGFP-N1 vector (between the *Eco*RI and *Bst*UI sites). Portion of the Kozak consensus translation initiation sequence (ACCATG) in the EGFP gene was further removed by site-directed mutagenesis (primer 5′-CGGGCCCGGGATCCACCGGTCGCCΔGTGAGCAAGGGCGAGGAGCTG, Δ = ACCATG) (QuikChange XL Site-Directed Mutagenesis Kit, Stratagene). The resulting pCMV/+801 construct (where +801 represents transcription start site of exon D) (Fig. 2A) was verified by DNA sequencing. The construct was predicted to encode an ORF-EGFP fusion protein containing 348 amino acids.

To construct pCMV/+1 (+1 representing transcription start site of exon D5), an *Xho*I site (underlined) was added to the 5′ end of +1 sequence by PCR using primer 5′-CGCCTCGAGTTTATGTCTATGAAGG. The *Xho*I-*Sac*I fragment containing *ATXN8OS* +1∼+848 sequences was inserted between the *Xho*I (in MCS of pEGFP-N1) and *Sac*I (in exon D of *ATXN8OS*) sites of pCMV/+801 to generate pCMV/+1. To construct pATXN8OS/−481, a 2.1-kb *ATXN8OS* gene 5′ fragment (AF252279 reversed complemented strand: 108333∼110454) was cloned by PCR and sequenced. The *Afl*III-*Sac*I fragment containing *ATXN8OS* −481∼+848 sequences was used to replace the corresponding fragment containing CMV promoter in pCMV/+801 to generate pATXN8OS/−481 (Fig. 2A). The *Afl*III-*Eco*RV fragment containing *ATXN8OS* −481∼−115 sequences in pATXN8OS/−481 was further removed to generate pATXN8OS/−114 (Fig. 2A).

### Real-time PCR Quantification of ORF-EGFP Transcripts

HEK-293 cells were plated into 6-well (6×10^5^/well) dishes, grown for 20 hr and transfected with the pCMV/+801, pCMV/+1, pATXN8OS/−114 and pATXN8OS/−481 constructs (4 µg/well). Forty-eight hours later, total RNA was extracted using the Trizol (Invitrogen). The RNA was DNase (Stratagene) treated, quantified, and reverse-transcribed to cDNA using High Capacity cDNA Reverse Transcription Kit (Applied Biosystems) with random primers. Using ABI PRISM® 7000 Sequence Detection System (Applied Biosystems), real-time quantitative PCR was performed on a cDNA amount equivalent to 250 ng total RNA with TaqMan fluorogenic probes Hs01382089-m1 (exon C2 and C1 boundary) for *ATXN8OS* and 4326321E for HPRT1 (endogenous control) (Applied Biosystems). Additional customized Assays-by-Design probe (forward primer: ACTGCATTTCAGGAGCAAAAAGAGA, reverse primer: GTCCCTGTGGTTTGAATCTATTCCA, TaqMan® probe: CAGTGGCCTCATTTTG) (*ATXN8OS* exon D5/D4 region, Applied Biosystems) was used for *ATXN8OS* mRNA quantification. Fold change was calculated using the formula 2^ΔCt^, ΔC_T_ = C_T_ (control) – C_T_ (target), in which C_T_ indicates cycle threshold. Statistical analysis of differences between the groups was carried out using one-way analysis of variance (ANOVA).

### FACS Analysis of ORF-EGFP Expression

HEK-293 cells were plated into 12-well (2×10^5^/well) dishes, grown for 20 hr and transfected with the above *ATXN8OS* ORF-EGFP constructs, pIRES2-EGFP and pEGFP-N1 (2 µg/well). Cells were harvested for fluorescence activated cell sorting (FACS) analysis. The amounts of GFP expressed were analyzed in a FACStar flow cytometer (Becton-Dickinson), equipped with an argon laser operating at 530 nm. A forward scatter gate was established to exclude dead cells and cell debris from the analysis. 10^4^ cells were analyzed in each sample.

### Confocal Microscopy Examination of ORF-EGFP Expression

HEK-293 cells were grown on poly-L-lysine (100 µg/ml, Sigma) coated coverslips (2×10^5^/12-well) and transfected with pIRES2-EGFP, pCMV/+801, pCMV/+1 and pATXN8OS/−114. After 48 hours cells were fixed in 4% paraformaldehyde for 15 min. Nuclei were detected using 0.05% DAPI (4′-6-diamidino-2-phenylindole). Cells were examined after mounted in Vectashield (Vector Laboratories Inc.) for GFP and DAPI fluorescence using a Leica TCS confocal laser scanning microscope optimized for simultaneous dual fluorescent imaging.

### Western Blot Analysis of ORF-EGFP Protein

Cells were lysed in RIPA buffer (10 mM Tris pH 7.5, 150 mM NaCl, 5 mM EDTA pH 8.0, 1% sodium deoxycholate, 1% NP-40 and 0.1% SDS) containing the protease inhibitor mixture (Sigma). After sonication and sitting on ice for 20 min, the lysates were centrifuged at 14,000 rpm for 30 min at 4°C. Protein concentrations were determined with the Bio-Rad protein assay kit, using albumin as standards. Proteins (25 µg) were electrophoresed on 10% SDS-polyacrylamide gel and transferred onto nitrocellulose membrane (Schleicher and Schuell) by reverse electrophoresis. After being blocked, the membrane was stained with anti-GFP (1∶200 dilution, Santa Cruz Biotechnology) or ORF antiserum (1∶200 dilution). The immune complexes were detected using horseradish peroxidase-conjugated goat anti-mouse (Jackson ImmunoResearch) or goat anti-rabbit (Rochland) IgG antibody (1∶10000 dilution) and Immobilon™ Western Chemiluminescent HRP substrate (Millipore).

### GST-ORF Construct and Antiserum

To construct GST-tagged ORF for antiserum production, *Bst*BI and *Eco*RI sites (italic) were added to the 5′ and 3′ ends of *ATXN8OS* ORF by PCR using primers 5′-GCGC*TTCGA* ***A*** **TG**TGCTTCACATCGAAGTC and 5′-CCG*GAATTC* **TCA**ACACTTCAACTTCCTATAC (initiation and termination codons in boldface). The 317-bp *Bst*BI-*Eco*RI fragment containing *ATXN8OS* ORF sequences was then inserted between the *Acc*I (location 928) and *Eco*RI (location 944) sites of pGEX-5X-3 (GE Healthcare). The location 928 *Acc*I site (italic) used was added by site-directed mutagenesis using primer 5′-GATCTGATCGAAG*GTCG* ***AC***GGATCCCCAGGAATTCC (mismatch nucleotides in boldface). The resulting pGST-ORF construct was verified by DNA sequencing and introduced into BL21(DE3)pLysS (Novagen). After IPTG induction, the 36-kDa antigen was purified using GST⋅Bind™ resin (Novagen) and used to raise antiserum in rabbit (LTK BioLaboratories).

### Lymphoblastoid and Neuroblastoma Cell Lines

Lymphoblastoid cells from a normal control were established (Food Industry Research and Development Institute, Taiwan) after obtaining informed consent. Cells were maintained in RPMI 1640 medium (GIBCO) containing 10% FBS. Human neuroblastoma SK-N-SH, SH-SY5Y and IMR-32 cells were cultivated in DMEM (IMR-32 and SK-N-SH) or 1∶1 mixture of DMEM and F12 medium (SH-SY5Y) containing 10% FBS.

### ORF Immune Detection

Cells (lymphoblastoid, neuroblastoma or HEK-293) or muscle tissue were lysed in urea lysis buffer (8 M urea, 4% CHAPS, 2% biolyte 3–10, 40 mM DTT). After ultrasonic homogenizing, protein extracts were centrifuged at 13,000 rpm at 4°C for 30 min and the supernatants transferred to new tubes. Pellets were then resuspended in SDS buffer (1.7% SDS, 20 mM Tris) by sonication. For Western blotting, proteins (30 µg) and aliquot of pellet were separated on 12% SDS-PAGE, blotted, stained with ORF antiserum (1∶200 dilution) or actin antibody (1∶10000 dilution, Chemicon) and immune complexes detected as described.

### ORF Identification

For 2D PAGE and 2D immunoblot, 5 volume of 9.8 M urea lysis buffer was added and aliquots of pellet suspension were first separated using Immobiline DryStrip (7 cm, pH 3–10) (GE Healthcare) and further separated by a 12.5% SDS-PAGE. The blotting membranes were stained with ORF antiserum (1∶200 dilution) or actin antibody (1∶10000 dilution, Chemicon) and immune complexes detected as described. The 2D gel was stained with SYPRO Ruby (Molecular Probe) and scanned on a Typhoon 9400 imager (GE Healthcare). The map was compared to the 2D immunoblot. The ORF-specific spots were punched out and subjected to reduction and alkylation by DTT/iodoacetamide, followed by in-gel digestion with freshly prepared Trypsin Gold (2.5 ng/µl, Promega) at 37°C for overnight. The obtained peptides were extracted with 50% acetonitrile containing 1% trifluoroacetic acid and tandem mass spectra were generated by liquid chromatography-mass spectrometry/mass spectrometry (LC-MS/MS) at Proteomics and Protein Function Core Laboratory, Center of Genomic Medicine, National Taiwan University. MS/MS data were searched using the Mascot search engine (www.matrixscience.com) in a database containing theoretical trypsinized fragments of 23-kDa ORF protein initiated at GUG^+953^ codon.
